# Agonists, Antagonists and Receptors of Somatostatin: Pathophysiological and Therapeutical Implications in Neoplasias

**DOI:** 10.3390/cimb46090578

**Published:** 2024-09-02

**Authors:** Argyrios Periferakis, Georgios Tsigas, Aristodemos-Theodoros Periferakis, Carla Mihaela Tone, Daria Alexandra Hemes, Konstantinos Periferakis, Lamprini Troumpata, Ioana Anca Badarau, Cristian Scheau, Ana Caruntu, Ilinca Savulescu-Fiedler, Constantin Caruntu, Andreea-Elena Scheau

**Affiliations:** 1 Department of Physiology, The “Carol Davila” University of Medicine and Pharmacy, 050474 Bucharest, Romania; 2 Elkyda, Research & Education Centre of Charismatheia, 17675 Athens, Greece; 3 Akadimia of Ancient Greek and Traditional Chinese Medicine, 16675 Athens, Greece; 4 Pan-Hellenic Organization of Educational Programs, 17236 Athens, Greece; 5 Department of Radiology and Medical Imaging, “Foisor” Clinical Hospital of Orthopaedics, Traumatology and Osteoarticular TB, 030167 Bucharest, Romania; 6 Department of Oral and Maxillofacial Surgery, The “Carol Davila” Central Military Emergency Hospital, 010825 Bucharest, Romania; 7 Department of Oral and Maxillofacial Surgery, Faculty of Dental Medicine, “Titu Maiorescu” University, 031593 Bucharest, Romania; 8 Department of Internal Medicine, The “Carol Davila” University of Medicine and Pharmacy, 050474 Bucharest, Romania; 9 Department of Internal Medicine and Cardiology, Coltea Clinical Hospital, 030167 Bucharest, Romania; 10 Department of Dermatology, “Prof. N.C. Paulescu” National Institute of Diabetes, Nutrition and Metabolic Diseases, 011233 Bucharest, Romania; 11 Department of Radiology and Medical Imaging, Fundeni Clinical Institute, 022328 Bucharest, Romania

**Keywords:** somatostatin, physiology, molecular effects, receptors, analogs, antagonists, octrotide, lanreotide, pasireotide, cancer

## Abstract

Somatostatin is a peptide that plays a variety of roles such as neurotransmitter and endocrine regulator; its actions as a cell regulator in various tissues of the human body are represented mainly by inhibitory effects, and it shows potent activity despite its physiological low concentrations. Somatostatin binds to specific receptors, called somatostatin receptors (SSTRs), which have different tissue distributions and associated signaling pathways. The expression of SSTRs can be altered in various conditions, including tumors; therefore, they can be used as biomarkers for cancer cell susceptibility to certain pharmacological agents and can provide prognostic information regarding disease evolution. Moreover, based on the affinity of somatostatin analogs for the different types of SSTRs, the therapeutic range includes conditions such as tumors, acromegaly, post-prandial hypotension, hyperinsulinism, and many more. On the other hand, a number of somatostatin antagonists may prove useful in certain medical settings, based on their differential affinity for SSTRs. The aim of this review is to present in detail the principal characteristics of all five SSTRs and to provide an overview of the associated therapeutic potential in neoplasias.

## 1. Introduction

Somatostatin (SST), also called Somatotropin-Release-Inhibiting Factor (SRIF) or Growth Hormone Release Inhibitory Factor (GHRIF) was originally discovered in 1973 [1], and occurs in two forms, SRIF-14 and SRIF-28. Both molecules originate from the same initial precursor molecule, called preproSRIF, via post-translational processing [2,3,4]. Both isoforms are expressed in the relevant tissues, although at this point whether they are secreted by the same or different cells has not been elucidated [5].

After the initial discovery of somatostatin, and the elucidation of its inhibitory action on growth hormone (GH) secretion [1], it was rapidly realized that it was also associated with a number of other physiological functions [6,7]. It was found that somatostatin also inhibited thyrotropin (TSH) and was capable of suppressing its secretion in TSH-producing tumors; moreover, somatostatin was capable of inhibiting adrenocorticotropic hormone (ACTH) release in conditions associated with increased secretion [8]. However, only after documenting the existence of different somatostatin receptors (SSTRs) [9], it became possible not only to begin identifying all the different effects of somatostatin—a process continuing to this day—but also to begin correlating them with differential receptor expression in different cells, tissues and organs.

Through its receptors, somatostatin exhibits mostly inhibiting effects, via decreasing intracellular calcium and cyclic adenosine monophosphate (cAMP), while increasing potassium outflow [10]. For this reason, it is generally regarded as an inhibitory peptide [11]. The actions of somatostatin are endocrine, paracrine, autocrine and neuro-modulatory [12,13,14,15]; in these roles, somatostatin is expressed and active in the central nervous system and the gastrointestinal system exhibiting a variety of effects [16,17,18,19].

Despite all the different and multifaceted actions and effects of somatostatin, it is practically useless in therapy due to its extremely short half-life [20]. This led to the need to develop SRIF analogs, with longer half-lives and, preferably, higher affinities for certain SSTRs, compared to the physiological endogenous peptide [21]. The analogs in clinical use today are octreotide, lanreotide and pasireotide, while other compounds are also under consideration [22]. On the other hand, somatostatin antagonists have also been synthesized, and are still the subject of experiments and animal studies [23,24].

In this review, we aim to thoroughly document the functions and distribution of all five SSTRs and to highlight the clinical relevance of each receptor in neoplasms. Subsequently, we will present the common somatostatin analogs used in clinical practice, as well as the current research status and future perspectives of somatostatin antagonists.

## 2. Description of Somatostatin Receptors

### 2.1. Overview of Somatostatin Receptors

The discovery of somatostatin is attributed to Brazeau and Guillemin [1] who isolated the then-unknown peptide from the ovine hypothalamus. It was a considerable time after that the five distinct SSTRs were identified, owing to the fact that radioligand studies allowed only for the distinction between SRIF binding sites and did not reveal the existence of five distinct receptors. The receptors are designated as SST_1_, SST_2_, SST_3_, SST_4_, and SST_5_, respectively [5,25].

According to the results of the SRIF-affinity studies, the five receptors can be classified into two groups, based mostly on their pharmacological and structural profiles. These are the SRIF-1 group (including SSTR_2_, SSTR_3_, and SSTR_5_) and the SRIF-2 group which includes SSTR_1_ and SSTR_4_ [21,26]. Despite their similarities, each receptor has different molecular weights and is coded for by different genes, with particular tissue distribution (Table 1), and functions (Table 2).

### 2.2. Structure and Properties of Somatostatin Receptors

All five of the SSTRs are G protein-coupled receptors (GPCRs), belonging to the rhodopsin family; in general, GPCRs act as receptors for extracellular stimuli, both physical and chemical, and their notable pathophysiological correlations make them prime targets for pharmacological interventions [61]. In accordance with the characteristic architecture of the GPCRs, all SSTRs share the common structure of seven transmembrane segments [62], and a sequence similarity of up to 57% [5]. However, their amino acid sequences differ, particularly at the level of the third intracellular loop as well as at the C-terminal end, leading to specific functional features [63].

Apart from the case of SSTR_2_, all SSTRs are coded from intronless genes. Regarding SSTR_2_, there exist two subtypes, SSTR_2A_ and SSTR_2B_, which are the product of alternative splicing; while both subtypes exist in rodents, only SSTR_2A_ is found in humans [64]. On the other hand, there are two SSTR_5_ variants in humans, produced by a different process [65]. Another particularity of SSTR genes is that they do not have TATA boxes [66], which are conserved sequences in the gene promoter region [67]. This may indicate that the genes themselves are insensitive to the knockout of their promoter-binding transcription factors [68].

A further common aspect of SSTRs is their association with G_i_/G_o_ proteins, which are part of the pertussis-toxin (PTX) sensitive protein family [37,69]. SSTR activation invariably leads to the reduction in intracellular cAMP and Ca^2+^, through inhibition of adenylyl cyclase. They can also cause inhibition of inward rectifier current K^+^ channels (Kir3.x) leading to hyperpolarisation and inhibition of intracellular Ca^2+^ entry through voltage-gated Ca^2+^ channels [70]; such actions inhibit hormone secretion [5]. The study of Pan et al. [71], also mentioned the role of protein tyrosine phosphatases in inhibiting cell proliferation, although the complete spectrum of molecular signaling associated with SSTR-induced proliferation inhibition is still poorly understood [5]. SSTRs can also form homodimers and heterodimers which can alter their pharmacological properties and signaling pathways [72]. They can also heterodimerize with other GPCRs leading to complex interactions and additional functional roles for the particular cells [73].

Receptor desensitization can occur for SSTRs similar to other GPCRs via phosphorylation and uncoupling from the proteins [74]. Subsequently, receptor internalization takes place, which is essential for the resensitization process [75]. The internalization process occurs at different concentrations of SST or SSTR agonist and in different conditions for each cell type and tissue [15,72,76,77].

### 2.3. Localization, Functions and Signaling Pathways of Somatostatin Receptors

#### 2.3.1. SSTR_1_

SSTR_1_, a highly glycosylated protein [31], is most prominently expressed in cells of the jejunum and the stomach [27]. It is also found in the CNS, concentrated in the presynaptic part of the neurons [28], where its activation leads to suppression of somatostatin secretion [78], of release-inhibiting hormones in general, and of growth-hormone-releasing hormone (GHRH) [79] and growth hormone (GH) itself [80] in particular. SSTR_1_ also acts as an inhibitory autoreceptor in the mediobasal hypothalamus, basal ganglia and retina [81]. Another role of SSTR_1_ is the regulation of the excitability of the cholinergic neurons of the forebrain [82]; an anti-inflammatory effect and a role in nociception have also been proposed [45,83,84]. The expression of SSTR_1_ in the β cells of Langerhans islets [85], most probably explains its role in regulating insulin secretion [86,87]. It is also found in the cell lining of veins and arteries [5].

Its signaling pathway, demonstrated to be highly influenced by the intracellular milieu, was found to be associated with G proteins both sensitive and insensitive to PTX [88,89,90,91,92]. Depending on the particular cell type, SSTR_1_ may exert its function via different signaling pathways [87,93,94]. SSTR1 activation has also been demonstrated to have cell migration-inhibiting properties, via inhibition of focal adhesion and actin stress fiber formation [95]. Activation of SSTR_1_ has also been shown to inhibit cell growth by tyrosine kinase dephosphorylation [96] and by p21 upregulation [97].

#### 2.3.2. SSTR_2_

SSTR_2_ is also highly glycosylated, and although two subtypes exist in nature, SSTR_2A_ and SSTR_2B_, only the first is expressed in humans [64]. It is commonly found in central and myenteric neurons, neuroendocrine cells of the gastric antrum, anterior pituitary cells and pancreatic islets [33,98,99,100] and also cells at the deeper layers of the cerebral cortex and in the cerebellum [101,102,103]. In general, SSTR_2_ is the most commonly detected SSTR [5], being also located in peripheral organs and structures [33,43,104,105]. Finally, SSTR_2_ is involved in a rather peculiar intracellular recycling pathway, in some cell lines, but the implications of this phenomenon are not fully understood [5]. This receptor is instrumental in the secretion of GH and thyroid-stimulating hormone (TSH) [50], is involved in inhibitory neuromodulation [5] and has a potential anti-epileptic action [102]. Feeding and drinking behaviors are also associated with SSTR_2_ [51,106] and the reduction in stress-induced or stress-related endocrine functions of the CNS and the pituitary gland [52,60]; an anti-depression role has also been proposed, associated with activation of SSTR_2_ and SSTR_3_, which in turn modulates serotonin release, although the precise mechanisms are still uncertain [47,48].

The signaling mechanisms are similar to that of SSTR_1_ and likewise, the effects are similar [5]. However, in some cell types, the inhibition of cAMP production by SSTR_2_ proved impossible [107]; this may be important in elucidating structural and functional differences in the morphofunctional differences of the G-protein-coupling domains of SSTRs. SSTR_2_ is involved in the regulation of the mitogen-activated kinase (MAP) kinase pathway in pituitary cells [108]; the tyrosine kinase pathway is also activated by SSTR_2_ [109,110]. The effects of SSTR_2_ activation are associated with cell cycle arrest and, by implication, inhibition of multiplication [111,112]. There is also a noteworthy interaction between SSTR_2_, in some cells, and Shank proteins, as mentioned by Günther et al. [5]; these proteins may be implicated in the regulation of synaptic transmission [113].

In some types of tumor cells, the activation of SSTR_2_ can trigger apoptosis [50]. It has also been reported that the dephosphorylation of SSTR_2_ is associated with reduced cell migration and invasion [114]. Notably, there is a difference in the genetic and epigenetic makeup of SSTR_2_ genes in some tumor cell lines [115,116]. The phosphorylation of specific residues of threonine and serine of this receptor at the carboxyl-terminal tail is related to its internalization in some cell types [117].

#### 2.3.3. SSTR_3_

SSTR_3_ is similar to SSTR_2_, being over 40% homologous; it is again heavily glycosylated in vivo [36]; despite its relative similarity to SSTR_2_, it is characterized by some unique structural features, compared to other SSTRs, such as the presence of a significantly longer carboxyl-terminal tail which lacks a potential palmitoylation locus [5]. The activation of SSTR_3_ results in adenylyl cyclase inhibition [118], potassium channel currents [119] and calcium channel currents activation [54]. In CHO-K1 cells, its activation also resulted in an increase in p53 and Bax levels [120], a fact that may prove useful in inducing apoptosis or preventing the growth of tumor cells. SSTR_3_ is also rapidly downregulated after prolonged agonist exposure, compared with other SSTRs [118,121]. The prime function of SSTR_3_ at a cellular level is hormone release inhibition; common examples include GH secretion inhibition [55] and insulin secretion inhibition [54]. Examples in rodents suggest that the presence of SSTR_3_ in brain nerve cells might be associated with memory and object recognition [122] and an anticonvulsant effect [123].

#### 2.3.4. SSTR_4_

This receptor is predominantly expressed in the brain and is found in a number of areas, such as the hippocampus, the hilar region of the dentate gyrus, amygdala, and hypothalamus [5]; its presence has also been ascertained in retinal ganglion cells [124]. It was also detected in pulmonary, heart and placental cells [125,126]. A host of other organs and structures are also characterized by SSTR_4_ expression [40]. Available data suggest that this receptor is not important in controlling the function of the anterior pituitary [127,128], but, based on mice studies, it is important in memory-related processes [58], locomotor functions [57,129], and stress-responses [130,131]. The activation of SSTR_4_, which in turn inhibits K_ir_3.x and voltage-activated Ca^2+^ channels is presumably associated with analgesic effects [132].

#### 2.3.5. SSTR_5_

The sequence of this receptor is less conserved among species compared to the other four SSTRs [5]. The signaling pathways of SSTR_5_ are common with those mentioned for other SSTRs, such as inhibition of cAMP [15,133,134,135,136]. However, this receptor is associated with a number of different signaling mechanisms which are dependent on the biochemical conditions in the environment of the receptor [15,136]. SSTR_5_ activation can lead to intracellular Ca^2+^ increase by activation of phospholipase C [137,138] but also to intracellular Ca^2+^ decrease by inhibition of voltage-dependent Ca_2_^+^ channels [139]. A number of specific pathways have been described by a number of researchers, including a cyclic guanosine monophosphate-dependent pathway, G_q_-mediated mitogen-activated protein kinase activation, and G_α_-mediated stress-activated protein kinases (SAPK)/Jun amino-terminal kinases (JNK) pathway activation [134,135,140,141,142,143]. In general, SSTR_5_ has an inhibitory activity on secretion [50,144,145]. SSTR_5_ also exists in Sertoli cells [146], and in aortic smooth muscle cells [147] and has a limited CNS distribution [5].

### 2.4. Importance of Somatostatin Receptors in Tumoral Pathology

The use of SSTRs as targets for pharmacological intervention has been considered extensively in the last decades. As shall be presented, especially so in the case of neoplasias, their expression patterns are important for prediction and treatment purposes. In general, it can be said that tumors having a low expression of SSTRs are less susceptible to therapy. Most tumors express more than one SSTR; however, there is usually a predominance of one subtype of receptor over the others and this might constitute a useful feature in both diagnosis and treatment.

#### 2.4.1. Somatostatin Receptors and Neuroendocrine Tumors

Neuroendocrine tumors (NETs) of the thorax are characterized by an increased SSTR_2_ expression [148], and sometimes of SSTR_5_ [149]. The expression of this receptor is also critical both for the diagnosis and treatment of small intestinal NETs and correlates positively with patient survival [150]. For NETs in general, Chang et al. [151] have argued in favor of a short-acting somatostatin diagnostic test. SSTR_2_ seems to be predominantly expressed also in the case of malignant insulinoma, although this did not predispose to a successful octreotide therapy [152]. Interestingly, in octreotide-treated patients, SSTR_2_ is internalized in the cell membranes of NETs [153]. Partial receptor internalization was noted in the case of pasireotide-treated patients [117,118].

The predominance of SSTR_2_ in the case of NETs has also been confirmed by other researchers [154,155,156]. In rectal NETs, an increased SSTR_2_ expression correlates with a better response to therapy and more favorable outcomes [157]. In more than half of NETs, SSTR_3_ expression can also be detected [158,159]; tumors from various regions, exhibiting neuroendocrine differentiation are not apparently characterized by elevated SSTR_3_ expression [160]. In the same manner, SSTR_2_ was the most important SSTR expressed in meningiomas [161,162], a fact that leaves open the exploration of the therapeutic potential of somatostatin analogs, at least in the case of inoperable metastatic tumors [163].

SSTR_2_ and SSTR_3_ were found to be expressed in pheochromocytomas and paragangliomas by Leijon et al. [164], with the other three SSTR receptors not being expressed at all; immunohistochemistry of SSTR_2A_ should be taken into consideration for personalized treatment schemes in paragangliomas [165]. SSTR_3_ was found to be the most expressed receptor in a study also of Parvizi et al. [166]; curiously, all SSTRs were found to be expressed at different rates in paraganglioma cells in the study of Kaemmerer et al. [167]. Nevertheless, the expression of SSTR_2_ renders paragangliomas, along with a host of other tumors, responsive to octreotide radioligand therapy [168].

In the case of acromegaly, SSTR_2_ is an important receptor due to its expression on adenoma cell membranes [169,170]. Therefore, the high expression of SSTR_2_ may contribute to the response to therapy [171,172,173]. Histologically, it is possible to divide adenomas into two different types, densely and sparsely granulated ones [174,175]; the first type, expresses SSTR_2_ much more than the latter and is thus more responsive to treatments with SSTR ligands [176,177]. It must be noted that, though not the most important SSTR in that case, SSTR_5_ appears to play a modulatory role; when expressed, it seems to upregulate the SSTR_2_-associated pathways, thus improving the therapeutical effectiveness, when ligands of SSTRs are used [44].

SSTR_2_ is also the one most predominantly expressed in thymic tumors [178]. The importance of SSTRs in thymic tumors is still a matter of debate, as there is a difference between in vitro cultured cells and in vivo thymomas; SSTR_3_ seems to be the only one expressed in both cases, while SSTR_1_ and SSTR_2_ were detected only in vivo [179]. In general, for thymomas, SSTR_3_ is the predominant receptor, and it is presumed that the absence of endogenous somatostatin, and hence its permanent inactive state, leads to uncontrolled proliferation [179].

In the cases of insulinomas [180], SSTR_2_ is the most commonly expressed receptor, although SSTR_3_ generally correlates with a larger tumor size and SSTR_3_ and SSTR_5_ co-expression correlates with a less positive outcome; in general, SSTR expression in insulinomas is weak, with about 20% of such tumors not expressing SSTRs at all [181].

In pituitary adenomas, which secrete GH, this receptor was found to be prominently expressed, along with SSTR_5_ and SSTR_2_ [36,182]; interestingly, in the case of pituitary adenomas secreting ACTH, SSTR_2_ expression was not usually detected [36,104]. In other pituitary tumors, only SSTR_3_ expression was notable [36,183,184].

In general, the available research data suggest that SSTR_4_ is not a common SSTR in neoplasias, although conflicting data regarding its expression on insulinomas exist [185,186]. The importance of SSTR_2_, and its common association with SSTR_5_ expression, was also highlighted in Merkel carcinoma cells [187,188,189]. The high SSTR_2_ expression has also been confirmed by Fagerstedt et al. [190], who have also noted its potential as a prognostic marker.

#### 2.4.2. Somatostatin Receptors and Squamous Cell Carcinomas

SSTR_5_ was found to be the predominant receptor in head and neck squamous carcinoma cells [191], whereas SSTR_1_ and SSTR_2_ were expressed at high rates and SSTR_3_ and SSTR_4_ were seldomly expressed [191]. Conversely, Lum et al. identified concomitantly high expression of SSTR_1_ and SSTR_2_ while SSTR_5_ expression was found to be particularly low [192]. In general, head and neck squamous carcinomas are characterized by a complex pathophysiology [193] and SSTR expression patterns and implications in the signaling pathways and carcinogenesis are not clear [194,195,196].

On a different note, the methylation of the SSTR_2_ genes was found to be a poor prognostic factor in the case of laryngeal squamous cell carcinoma [116]; the expression of SSTR_2_ in the case of nasopharyngeal carcinoma was found to be moderate to high [197]. The importance of this receptor and its associated signaling, expression and related epigenetics in squamous cell carcinomas of the head and neck has represented a research interest for Fan et al. [198]. SSTR_2_ and SSTR_5_ expression were found to be high in the case of uveal melanoma cells [199]. Based on the study of Valsecchi et al. [200], it is possible to use SSTR expression both therapeutically and to estimate survival rates. 

#### 2.4.3. Somatostatin Receptors and Reproductive System Carcinomas

In breast cancer, SSTR_1_, SSTR_2_ and SSTR_3_ were highly expressed, with SSTR_4_ and SSTR_5_ at comparatively lower percentages, but still over 60% [201]. Another study [202], found SSTR_1_ expression to be pronounced, followed by SSTR_4_ and the rest at considerably lower levels. In the case of ovarian cancer, one study [203] determined that the expression of SSTR_1_, SSTR_2_ and SSTR_5_ was predominant, while another [204] found SSTR_3_ expression to be higher. In general, somatostatin expression and its clinical significance in ovarian tumors is a matter requiring further research [205]. A recent study, by Zhao et al. [206], found SSTR_5_ to be the receptor most expressed in ectopic endometrial tissue, followed by SSTR_2_ [207]. The treatments currently available are limited and do not target SSTRs [208].

For prostate cancer, SSTR_1_ was found to be the primary receptor expressed in the case of prostate neoplasias, amongst 80 different samples [209]; this overexpression of SSTR_1_ has been also documented by other researchers [210,211]. There it inhibits the production of prostate-specific antigen (PSA) and has antiproliferative effects [212]. Another study correlated the increased expression of SSTR_1_ with increased tumor suppression [213]. However, overall, SSTR expression is relatively low in prostate neoplasia, with SSTR_5_ and SSTR_2_ being expressed at a low percentage of about 10% [214,215].

#### 2.4.4. Somatostatin Receptors and Digestive Carcinomas

In gastric cancer, SSTR_2_ is the most predominant [216], while SSTR_3_ seems to be more expressed, on average, in healthy and not in cancerous gastric mucosa [217]. It was also found that the expression of SSTR genes was inversely proportional to metastatic potential [218]. The expression of SSTR_2_ in colorectal cancer cells can be used as a prognostic marker, according to the study of Casini Raggi et al. [219], although it was noted that SSTR_2_ expression between healthy and cancer cells was not that much different.

SSTR_5_ was found to be expressed more than all other SSTRs in MALT-type lymphomas, and to be more expressed in gastric-type lymphomas, along with SSTR_3_ and SSTR_4_, compared to extragastric ones; in addition, the presence of SSTR_5_ in lymphomas was found to correlate with a more favorable prognosis [43].

## 3. Therapeutical Uses of Somatostatin Analogs in Neoplasias

In general, the activation of SSTRs, in the case of neoplasias, exerts effects at a cellular level—antiproliferative [220,221,222], apoptotic [223,224,225]—and at a systemic level—antineoplastic [45,226,227] and anti-inflammatory [22,228,229,230]. Therefore, the use of somatostatin analogs has seen widespread use, in different neoplastic syndromes; on the other hand, taking advantage of the inhibitory effects of somatostatin on hormone and bioactive molecules secretion, some use of somatostatin analogs has demonstrated promising effects in other pathological conditions. 

The primary reason for using somatostatin analogs instead of somatostatin itself is that endogenous somatostatin is very rapidly degraded by the human body [5]; the degradation of somatostatin is performed by ubiquitous plasma and tissue peptidases [231]. Another problem with somatostatin use was revealed when attempting to manage acromegaly, the initial disease where somatostatin and its analogs saw therapeutic application; the suppression of GH secretion was accompanied by an unacceptable suppression of insulin secretion [232,233,234,235]. At first, it was difficult to explain how and why somatostatin analogs inhibited GH secretion more than insulin secretion, a conundrum only solved after the existence of different SSTRs was realized [9]. Current research interests include the identification of SSTR pathways, and their interference with physiologic regulation mechanisms [5]. This is an important parameter especially in the design of analogs for therapeutic purposes.

At some level, all somatostatin analogs mimic the actions of somatostatin, and this is their common pharmacodynamical property. However, they preferentially act on different receptors (Table 3), and therefore it is possible to choose different analogs to take advantage of the differential expression of SSTRs in different tumors. For example, while endogenous somatostatin has its greatest selectivity for SSTR_3_, with a Ki of about 6 nM, octreotide has a Ki of over 1000 nM for SSTR_1_ and SSTR_5_, lanreotide has a commensurate selectivity only for SSTR_1_, and pasireotide has a Ki of >100 nM for SSTR_4_ [9,92,236,237,238].

Of the existing somatostatin analogs, only three have been approved for clinical use, octreotide, lanreotide and pasireotide (Table 4); the two former are first-generation analogs, and the latter is a second-generation analog [239]. Octreotide, a cyclic octapeptide, is more stable from a metabolic point of view, compared to somatostatin, because of its D-confirmation of amino acids and the presence of a disulfide bridge [240]—these increase its resistance to degradation by intestinal peptidases [241] Nevertheless, it retains the basic Phe-Trp-Lys-Thr motif of somatostatin [242]. The secondary structure of lanreotide is essentially the same, but there are some differences in amino acids compared to octreotide [243]. Pasireotide is completely different in terms of composition and structure from the other two analogs, being a six-membered homodetic cyclic peptide composed from L-phenylglycyl, D-tryptophyl, L-lysyl, O-benzyl-L-tyrosyl, L-phenylalanyl and modified L-hydroxyproline residues joined in sequence [244]

Regarding the rest, vapreotide has had some applications in humans, animals and in vitro [245,246,247,248,249,250,251,252,253,254,255,256,257], but it has had limited application in neoplasias as of yet [258,259,260]. Veldoreotide is a recently synthesized analog and little research exists on its potential applications [261]. Finally, seglitide, existing since the 1980s [262], has not been widely used, with a few exceptions [9,246].

**Table 3:** Pharmacological properties of clinically used somatostatin analogs [263,264,265,266,267,268].

Somatostatin Analog	Octreotide	Lanreotide	Pasireotide
Most effective administration route	Subcutaneous	Subcutaneous	Subcutaneous
Cmax attainment	30 min (subcutaneous) 1.67–2.5 h (oral)	n/a	0.25–0.5 h
Volume of distribution (V_L_)	13.6–30.4 L	15.1 L	>100 L
Protein binding	65%	n/a	88%
Metabolism	Liver	Gastrointestinal tract	Liver
Half-life	2.3–2.7 h	22 d	12 h
Clearance	7–10 L/h	23.1 L/h	7.6 L/h
Elimination	Urinary (32%) Faecal (30–40%)	Urinary (<5%) Faecal (<0.5%)	Hepatic (48.3%) Urinary (7.63%)

**Table 4:** Use of somatostatin analogs in the treatment and management of various conditions.

Somatostatin Analog	Condition	Tumor-Expressed SSTRs	References
Octreotide	Acromegaly	SSTR_2_, SSTR_5_ (minor role)	[269,270]
	Congenital hyperinsulinism	SSTR_2_	[271]
	Thymic tumors	SSTR_2_ (minor role), SSTR_3_	[178]
	Lymphoma (potential treatment)	SSTR2	[272]
	Neuroendrocrine tumors	SSTR_2_, SSTR_5_ (minor role)	[273]
	Merkel cell carcinoma	SSTR_2_, SSTR_5_	[274,275]
	Hepatocellular carcinoma	All SSTRs	[21]
Lanreotide	Acromegaly	SSTR_2_	[34]
	Neuroendocrine tumors	SSTR_2_, SSTR_5_ (minor role)	[273,276,277]
	Hepatocellular carcinoma	All SSTRs	[278]
Pasireotide	Acromegaly	SSTR_2_ (minor role), SSTR_5_	[34,279]
	Congenital hyperinsulinism (proposed) and drug-induce hyperglycaemia in Cushings syndrome	SSTR_5_	[271,280]
	Neuroendrocrine tumors	SSTR_1_, SSTR_2_, SSTR_3_, SSTR_5_	[281]

Despite the numerous potential uses of somatostatin analogs outlined above, their use is not without a risk of side-effects ranging from mild to severe and even life-threatening. Frequent side-effects may be localised (local infusion reactions, pain and swelling), nervous system-associated (headache, nausea, or vomiting), gastrointestinal (abdominal pain/bloating, diarrhea, and gallstone formation—the frequency of that depends on the presence of other comorbidities), and appetite loss. Other effects such as arrhythmias and/or bradycardia, hypertension, vitamin B_12_ deficiency, impaired glucose tolerance (depending on comorbidities) and fatigue/malaise are rare; finally, the very rare side effects comprise hypothyroidism, hepatic injury and pancreatitis [266,267,268,282].

For example, as analyzed in the following parts, in conjunction with octreotide, octreotide-induced hypoglycaemia, in patients with insulinomas, may even prove fatal [283]; likewise, the gallstone incidence increase may be so high, that even precautionary surgical removal of the gallbladder may be recommended [284].

### 3.1. Therapeutical Uses of Octreotide in Neoplasias

Octreotide was first synthesized in 1979 by W. Bauer [285], and compared to somatostatin, it is an even more potent inhibitor of glucagon, insulin and growth hormone release [14]. While initially reported as carrying a potential analgesic effect in cancer patients, this was disproved in a clinical trial [286]; another study reported that it may be beneficial in cases of chronic non-malignant pancreatic pain [287]. Today, octreotide is the oldest somatostatin analog in clinical practice, being used for over 40 years [288].

In general, octreotide inhibits growth hormone and glucagon, various gastrointestinal hormones and reduces splanchnic blood flow through mechanisms similar to somatostatin, but with higher effectiveness [264]. Common side effects comprise the reduction in gall bladder contractility, thyroid stimulating hormone release [264] and B_12_ vitamin levels [266]. Notably, octreotide can be found in breast milk, in high concentrations [289,290], but this is not considered very dangerous for the infant given the low oral absorption of the drug [263]. Extreme caution is required if and when octreotide is administered in elderly people, as it decreases hepatic and renal and even cardiac functions [291]. To our knowledge, there are no reports in scientific literature regarding octreotide overdose. On the other hand, octreotide administration is effective in treating sulfonylurea overdose [292,293,294,295]; this drug is the oldest antidiabetic medication, dating back to the 1950s [296], and is still used by type 2 diabetes patients [297,298,299,300].

A case report, regarding insulin glargine overdose in a type 2 diabetes patient [301], corroborates the potential of octreotide to prove useful in treating antidiabetic drug overdose. Finally, it has been theorized that octreotide could be administered to treat drug overdose in general [302]. However, alongside the therapeutical merits of octreotide, the cessation of its administration, especially if it is prolonged, may be associated with significant side effects [303,304,305].

Subcutaneous administration is the most effective, with virtually complete absorption, [263] while oral administration was found to be less efficient by approximately 30% [266]. Oral octreotide administration must be performed on an empty stomach, as the presence of food reduces the absorption of octreotide by about 90%. This is important in the context of bioavailability. For example, the action of octreotide on tumor cells typically extends for between 8 and 12 h post-administration [291].

#### 3.1.1. Octreotide in the Treatment of Thymic Tumors

Thymic tumors are very rare, and they comprise a group of different histological patterns, which are in turn associated with different survival rates [178]. In general, in thymic cancers, more than one SSTR is present, with the expression of SSTR_2_ being predominant in the majority of cases [178]. Octreotide has been shown to regulate the development and maturation of thymocytes [306,307] and it is worth mentioning that the activation of SSTR_1_ and SSTR_2_ promotes the proliferation and maturation of thymocytes in fetuses [308,309]—this is one of the very few non-inhibitory actions of somatostatin in the human body. Completely different from the pattern of healthy cells, thymomas were found to mostly express SSTR_3_. In general, the use of octreotide to combat thymic tumors can be regarded as promising, based on a number of case studies [310,311,312,313,314,315,316] and case series [317,318,319]. However, reported administered doses vary significantly from 20 mg per month for long-acting release forms up to 200 mg three times per day [314,316], With various dosages available and the ensuing difficulty in assessing the side effects it is recommended for octreotide to be used only in cases where significant octreotide uptake by the tumor cells can be demonstrated [178,314].

#### 3.1.2. Octreotide in the Management and Treatment of Neuroendocrine Tumors (NETs)

Neuroendocrine tumors (NETs) are a group of heterogeneous neoplasms associated with cells of the diffuse neuroendocrine system (DNES); different NET classification schemes have been proposed [320,321,322]. Regardless, their clinical significance very much depends on their site of origin [273]. Crucially, NETs are known to have a pronounced expression of SSTRs [282]; in most cases, SSTR_2_ is the most prominently expressed, at least in gastroenteropancreatic NETs [323]. In general, long-acting somatostatin analogs present many advantages in treating well-differentiated NETs [324]. Personalized NET treatment with somatostatin analogs presents one of the challenges of modern personalized medicine [325]. Identification of adequate biomarkers is necessary for refining personalized treatment in NETs, and microRNA may be useful response predictors and prognostic markers [325]. Reported dosages of long-acting analogs vary between 20 and 180 mg/28 days [324]. Recent research indicated that there are no substantial gender differences in response to somatostatin analogs treatment in NETs, despite early evidence to the contrary from preclinical studies [326].

Octreotide with its high affinity for SSTR_2_ can be effectively used to manage the symptoms associated with NETs, as evidenced by some relevant studies [284,327,328]. NETs are associated with the so-called carcinoid syndrome, frequently caused by the release of serotonin, and bioactive peptides by these tumors [329]; common symptoms comprise flushing and diarrhea, but atypical symptoms, such as pellagra and bronchospasms, have also been recorded [330]. In such cases, octreotide may be used to manage carcinoid crises or as prophylaxis [331], although there is no consensus as to its effectiveness [332,333]. Also, the heterogeneity as well as the absence of a sufficient amount of clinical data present therapeutic challenges in NETs [334].

A testament to the importance of SSTR_2_ in the case of NET treatment is the case of advanced insulinomas, which typically have a low SSTR_2_ expression and are therefore less or non-responsive to octreotide treatment, demonstrating that SSTR expression is a critical factor in treatment effectiveness [273]. In addition, octreotide treatment can lead to hypoglycemia, possibly because of the independent downregulation of glucagon [284]. It is also possible to use octreotide to reduce gastrin secretion in the case of Zollinger-Ellison syndrome [335]; this may be localized or associated with multiple endocrine neoplasia type 1 [336]—presumably, in this latter case octreotide or some other somatostatin analog could be used target multiple neoplasias. 

In the case of neuroendocrine carcinoma (NEC) of the lung, as with various neuroendocrine tumors, they frequently express more SSTRs compared to normal tissues [337], and are thus susceptible to somatostatin action [338]. This strategy is frequently sufficient for symptom relief—symptoms arising from the overproduction of hormones from the tumors—but can rarely reduce tumor size [339]. But for any targeted somatostatin therapy, the precise pattern of SSTR expression of the tumor must be known [340]. This analysis may be performed by a variety of methods, mainly immunohistochemical [341,342,343,344,345,346,347,348], and is essential, in the case of all therapeutical somatostatin analog use and not just for octreotide. 

For lung NECs, the predominant SSTRs are SSTR_2A_ and SSTR_1_, with SSTR_4_ and SSTR_5_ less frequently expressed [349], depending on the degree of differentiation of the tumor [337,346]. In the case of small cell lung cancer, a type of high-grade neuroendocrine cancer [350], the presence of SSTRs may render them responsive to somatostatin analog treatment, although it has, as of yet, no known predictive value [351]. The determination of SSTR expression may be possibly performed in the future with next-generation sequencing as proposed by Kruglyak et al. [352] and Cainap et al. [353]; in fact, such sequencing could be applied to most tumors [354], specifically to look for SSTR expression or mutations.

Based on current evidence, it is possible that in slow-growing diseases, treatment with somatostatin analogs is not really more effective than the “watch-and-wait” approach [273]. This was noted in 1996, by Perry and Vinik [355], who commented that for slow-progressing tumors, it was difficult to assess the effectiveness of octreotide therapy on tumor growth; nevertheless, there was a clear therapeutical effect, in cases of metastatic tumors. On the other hand, again taking advantage of the increased expression of SSTRs observed in NETs, radiolabelled octreotide can be used for systemic radiotherapy, to deliver radioisotopes specifically to tumor cells [356,357,358,359,360]. At any rate, the administration of octreotide, or lanreotide, is recommended pre-operatively, to manage hormonal imbalances caused by the NET, before its removal [361]. Caution is necessary in the case of insulinomas, where octreotide administration may even be fatal [283].

Apart from therapy per se, octreotide can be used for nuclear imaging purposes, in order to ascertain the SSTR expression of NETs, while it also enables the identification of possible occult tumors [362,363]; the most sensitive method available, making possible the detection of very small tumors is Positron Emission Tomography (PET) with gallium 68–labelled octreotide [364,365]. The sensitivity of PET in NETs is over 79% [366,367,368], except for insulinomas, where the 25% sensitivity [369] may be explained by the aforementioned diminished SSTR_2_ expression. The use of such sensitive methods is instrumental in the detection and staging associated with a more aggressive″ approach [361]. Such considerations aside, it was determined that in the case of imaging for neuroendocrine tumors, octreotide, and somatostatin analog treatments in general, do not seem to affect imaging in a negative way [370,371,372].

#### 3.1.3. Octreotide in the Treatment of Lymphomas

Lymphomas are a diverse group of malignancies, generally arising from the clonal proliferation of lymphocytes [373,374]. The team of Dalm et al. [375] used octreotide in quantitative reverse transcription polymerase chain reaction autoradiography and determined that, in their study group, the expression of SSTR_2_ and SSTR_3_ seemed to be rather low. 

The lower SSTR expression in lymphomas, at least compared to NETs, was corroborated by the subsequent research of Ferone et al. [376]. However, from a diagnostic point of view, the differential expression of SSTRs can be used to differentiate extragastric from gastric MALT-type lymphomas and is also a useful tool for staging the tumors and giving a prognosis [377]. On the other hand, there is a discussion regarding the higher specificity but lower sensitivity of octreotide scintigraphy, compared to more traditional methods of tumor imaging [378].

Apart from the aforementioned diagnostic value, studies mention that the expression of SSTRs may be used for treatment, in those lymphoma types that highly express SSTR_2_ [272]. But, for octreotide in particular, or indeed any somatostatin analog in general, to be useful therapeutically, the precise pattern of SSTR expression in lymphomas must be elucidated [205].

#### 3.1.4. Octreotide in the Treatment of Merkel Cell Carcinoma

This is a rare, highly aggressive endocrine malignancy of the skin, with a mortality rate higher than that of melanoma [379,380,381]. As mentioned in the previous sections, Merkel carcinoma cells preferentially express SSTR_2_ and SSTR_5_ [188], meaning that in theory octreotide must be effective in such neoplasias. A treatment scheme of avelumab and octreotide, in a single patient, in a recent case report proved successful [274]. The subsequent study of Akaike et al. [275], who used somatostatin scintigraphy and then an octreotide-based treatment exhibited somewhat positive results; the promising potential of somatostatin analogs in Merkel cell carcinoma is also supported by the research of Anderson et al. who treated patients with monthly intramuscular injections of 20–30 mg with no significant adverse effects [189].

#### 3.1.5. Octreotide in the Treatment of Hepatocellular Carcinoma (HCC)

Hepatic cancer is amongst the leading types of cancer at a worldwide level [382], with surprisingly high recurrence rates [383,384,385]; this is attributable to a variety of causes [386]. HCC, the most frequent hepatic cancer, is associated with a variety of risk factors [21,387,388]; its treatment or even management is exceedingly difficult, often requiring a complex approach [389,390,391]. Crucially for somatostatin analog treatment, hepatocellular carcinoma cells express all five SSTRs, which are not expressed in physiological hepatocytes; apart from HCC treatment, somatostatin analogs may also be used in cirrhotic liver patients [228,392], and have been successful in treating associated ascites [287,393,394].

Octreotide has been used extensively in the treatment of HCC, in numerous clinical trials. Although a number of such trials had a positive outcome [278,395,396,397,398,399,400,401,402,403,404,405,406,407,408,409], an equally substantial number of trials had a negative result [410,411,412,413,414,415,416,417]. The mixed results of the clinical trials may be due to the heterogeneity of the different studies; additionally, it must be stressed that many of the negative results are from trials involving end-stage patients [21]. Octreotide was administered in various forms including subcutaneously (150–750 μg/day) and intramuscular (30–40 mg/month).

### 3.2. Therapeutical Uses of Lanreotide in Neoplasias

Much like octrotide, lanreotide antagonizes hormone secretion and also has antiproliferative effects; it has high affinity for SSTR_2_ and SSTR_5_. Not so much data are available compared to octreotide, a fact consistent with the more limited use of lanreotide. The reported side effects are gastrointestinal, namely diarrhea, abdominal pain, nausea, vomiting, abdominal distention, impaired glucose tolerance, and gallstone formation, but also include cardiovascular side effects, alopecia, and pain and irritation at the injection site. Not all of these effects occur with the same frequencies and has not been established if and how much they are dose-dependent [268]. Lanreotide is not recommended during breastfeeding, although it is in case unlikely to reach toxic levels in the infant serum [418].

Just like octreotide, lanreotide can be used for the management of symptoms associated with NETs [273,419,420]. A clinical trial conducted by Caplin et al. [277] determined that the patients receiving lanreotide treatment (subcutaneous 120 mg/month aqueous-gel injection) had a better outcome, compared to placebo-receiving patients. Lanreotide autogel, is the approved initial treatment for patients with unresectable low-grade metastatic malignant insulinomas, effectively managing tumor growth and leading to hypoglycemia control in some but not all cases [276]. Also, a single case study [421] supports the use of lanreotide in duodenal neoplasm patients.

It must be noted that based on recent results, a half-year treatment with lanreotide in NET patients, did not significantly alter the patient’s perception of the disease [422]. However, NET patients seem to prefer lanreotide to octreotide in that it is easier to administer and is associated with fewer adverse reactions at the injection site [423]; another study also noted that satisfaction of patients with their lanreotide treatment regimen [424]. On the other hand, the randomized trial of Raj et al. [425] did not find significant differences between the preferences of NET patients for octreotide and lanreotide. Finally, in the treatment of hepatocellular carcinoma, the results of lanreotide administration were mixed, with one positive trial, in association with octreotide, [278] and one negative trial [410]; lanreotide is considered an overall safe drug [426,427] although adverse reactions have been cited, most recently after the administration for a pancreatic NET resulting in pituitary apoplexy [428].

### 3.3. Therapeutical Uses of Pasireotide in Neoplasias

This is a somatostatin analog activating most SSTRs, and having the lowest affinity for SSTR_4_. In addition to the hormones inhibited by octreotide and lanreotide, it also inhibits ACTH secretion [267]. Its side effects are very similar to those of lanreotide; while not likely to pass into breastmilk, lactating mothers are nonetheless advised to substitute it with an alternative treatment [429].

Pasireotide, being a newer drug, has not been extensively tested against NETs, but for a couple of clinical trials, such as Bruns et al. and Kvols et al. [281,430] which showed promising preliminary results. Given that desensitization to octreotide and lanreotide is a well-documented phenomenon [431]—possibly due to internalization or downregulation of SSTR_2_ in target cells, or overexpression of other SSTRs [432,433,434]—pasireotide, with its increased affinity for all SSTRs except for SSTR_4_, may offer a good alternative therapeutical approach [281]. It must be noted that perioperative treatment with pasireotide decreased the risk of significant pancreatic fistulas after pancreatic resection [435]. In general, the expression of SSTR_2_ by carcinoid tumors may allow for low doses of pasireotide, which, while still effective, will not be associated with significant side effects [436]. In another neoplasia, HCC, pasireotide was found to be ineffective [437,438].

## 4. Somatostatin Antagonists: Current Evidence and Studies

The role of somatostatin antagonists mainly revolves around radio imaging, based on the early concepts developed for single-photon emission computed tomography (SPECT) and PET [439]. Already SSTR imaging is a promising aspect in thyroid cancer [440]; SSTR-based imaging for neuroendocrine neoplasms is already an established approach [441], and generally, SSTR-based imaging can be used to monitor response to therapy and plan further therapeutic approaches [442]. Numerous radioligands have been developed that bind to the SSTR receptors providing essential anatomical and functional information through hybrid imaging, while also allowing for concurrent treatment via the so-called peptide receptor radionuclide therapy [443]. The precursors of most SSTR-targeting radiopharmaceuticals are the corresponding SSTR-targeting drugs already discussed; for example, the conversion of octreotide from agonist to antagonist is simple, involving just two positions [444].

Poorly internalized somatostatin antagonists are more effective for imaging, compared to highly internalized agonists [445]. Furthermore, it is believed that, at least in the case of some neuroendocrine tumors, the use of antagonist radiotracers may be more effective than the use of agonist radiotracers, as they can bind to a larger number of SSTR conformations [446]. These encouraging results were corroborated by the subsequent study of Fani et al. [447]. In treatment, the data on the use of SSTR antagonists are limited. There exist a few antagonists that have been tested for such purposes, with different affinities for some or all of SSTRs.

One characteristic case is the research of Modarai et al. [448] on colorectal cancer; they hypothesized that SSTR_1_ was instrumental in maintaining the quiescence of colonic stem cells; their abnormal proliferation leads to colorectal cancer. In an in vitro study, they determined that somatostatin was not expressed in cancer cells, in contrast with SSTR_1_ which was expressed. Regardless, the precise role significance of certain SSTRs in colorectal cancer, when correlated with the local microenvironment and microbiome has not yet been elucidated [449]. The use of cyclosomatostatin was important in differentiating between the different mechanisms controlling the multiplication and differentiation of stem cells [448]. Up to this point, cyclosomatostatin has been used in a number of other animal experiments [450,451,452,453,454,455,456,457,458,459,460]; to our knowledge, no clinical use of it has yet been made.

Recent studies explore novel compounds with therapeutic aims, and their area of relevance might be expanded to cancer treatment [461,462,463]. Following up on the results of Sprecher et al. [464], the team of Hirose et al. [465] developed and tested a 5-oxa-2,6-diazaspiro [3.4]oct-6-ene derivative, which is a novel SSTR_5_ antagonist, effective against type 2 diabetes mellitus in mice, as it augmented glucose-dependent insulin secretion. Another antagonist, CYN 154806, a solid cyclic octapeptide, has been successfully tested in vitro [466] and in vivo in mice [467]. The SSTR_3_-selective MK-4256, has been successfully tested and is able to reduce glucose levels in mice [468]. PRL 2915, with high affinity for SSTR_3_ and a lower affinity for SSTR_5_ [469,470], has been successfully applied in vitro to stop aortic ring contractions [470]. BIM-23056, a linear octapeptide with SSTR affinities similar to that of the previous antagonist, has not been very successful in vitro, as of yet [471]. Finally, another novel somatostatin antagonist, a spiropiperidine analog, proposed by Liu et al. [472] may prove useful in the management of diabetes mellitus, if optimized to achieve the necessary pharmacokinetic profile.

## 5. Discussion

SSTRs have numerous important physiological functions, which depend on their associated signaling mechanisms and distribution in the human body. The differential distribution of SSTRs in cancers enables the selective use of somatostatin analogs, as viable solutions in the treatment of neoplasias or associated symptoms, with reasonably few side effects, in many cases. From the three analogs currently in clinical use, octreotide, which has been in constant use for over four decades now, is characterized by ample clinical data both regarding its effectiveness and side effects; for the other agonists and antagonists, evidence is encouraging but fewer in comparison.

Apart from the cases of somatostatin agonists and antagonists presented above, there are also other compounds, whose application may provide new insights. Such an example is dopastatin, a chemical compound combining the structure of dopamine and somatostatin; the use of this compound was envisaged as a therapeutic agent in patients with acromegaly, and indeed, its ability to act both on SSTR_2_ and dopamine D_2_ receptors was found to greatly enhance its effects [473,474]. While this enhanced effect has not been yet explained, Rocheville et al. [475] note that it is possible to attribute it to a heterodimerization between the two aforementioned receptors. Based on these data, it is possible that, in the future, chimeric compounds combining somatostatin with other compatible hormones, could be used, especially in the case of multi-hormone-dependent tumors, such as breast cancer and prostate cancer [476] and ovarian cancer [477,478]. Combination therapy of somatostatin agonists and various chemotherapeutic drugs is being researched for a variety of cancers, including NETs, with some favorable results regarding response rates and progression-free survival [479].

The use of antagonists in combination with radioisotopes, for cancer therapy is another opportunity, given the high efficiency of antagonists in binding to specific receptors, as demonstrated in the relevant imaging studies. Such a type of therapy was the subject of the research of Kong et al. [480], who achieved good results in patients with bulky neuroendocrine tumors. In cancers, the main application of agonists is to inhibit hormone secretion and exhibit antiproliferative effects through cell cycle arrest and pro-apoptotic pathways; conversely, antagonists may demonstrate antitumoral effects by increasing the secretion of certain growth-inhibiting factors and pro-apoptotic cytokines as well as through the disruption of the tumoral adaptive mechanisms with secondary restoration of tumor sensitivity to the treatment [481].

Based on the aforementioned research results, and regarding the distribution of SSTRs in different pathologies, it can be seen that in some neoplasias, there is differential SSTR expression which may be important for staging, prognosis and therapy. Nevertheless, there are some cases where, for the same type of cancer, there are conflicting research results. As such, there are still certain cases where the presence of SSTRs and their relative expression has not been definitely assessed as to their significance and the associated therapeutic potential.

The study of Angelousi et al. [482], focused on the resistance patterns of NETs to somatostatin analog treatment, highlighting the considerable diversity of tumor resistance, which is presumably associated with the particular molecular and genetic mechanisms of each different tumor. Such data are indicative of the need to personalize treatments based on differential tumor parameters and responses. Other important issues in somatostatin analog treatment are the determination of the most effective regimens as well as whether dose-dependent effects occur [483,484,485].

Apart from the neoplastic syndromes mentioned in our analysis, it is possible that other neoplasias may be characterized by SSTR expression; this could be the focus of future research. Moreover, and taking into account the known resistance patterns to classic somatostatin analogs and their side effects, the introduction of other, non-peptide, SSTR agonists, such as the compound used by Juliana et al. [486], may improve the current treatment schemes. The treatment of neoplasia patients is oftentimes a complicated issue [487], as seen in the case of NET patients, and, especially when it includes somatostatin analogs, should be performed at dedicated and experienced centers [488]. Moreover, numerous alterations within the tumor microenvironment may be reflected by serum changes of various metabolites or cell expression, leading to further interferences between carcinogenesis and the immune response [449,489,490,491].

Interestingly, despite the known side effects of somatostatin analogs, an increased percentage of acromegaly and NET patients are still given the maximum allowed analog dose, at least in some regions [492]. It has also been found, in acromegaly patients, that prolonged octreotide treatment does not seem to increase *Helicobacter pylori* incidence [493] and despite earlier ambiguous research results [494], it was even recently demonstrated, in rats, that octreotide treatment can protect against *H. pylori*-induced gastritis [495]. Although there is no direct link between acromegaly and gastritis, it was observed that octreotide treatment was associated with increased gastritis incidence, and it was believed that *H. pylori* might represent a causative factor [493,494].

Apart from the administration of somatostatin analogs, another avenue that should be considered is the induction of endogenous somatostatin production. It has been demonstrated that systemic capsaicin administration can cause the elevation of somatostatin levels [496,497,498,499]. This might hold therapeutical potential, perhaps by local instillation of capsaicin, thus obviating the risk of capsaicin side effects that accompany its systemic administration in high doses [500,501,502,503,504,505]. The interplay with SSTRs and somatostatin levels in cancers is of interest since capsaicin itself also exhibits antitumoral effects [506,507,508,509]. Another possibility is the use of nanoparticles, which are already been studied for the deployment of anti-cancer agents [510,511,512,513,514], or lipoparticles [515,516] for targeted delivery to neoplasias.

Modulation of endogenous somatostatin production might be achieved through acupuncture, and literature data showcase the effects of acupuncture on serum hormone and protein levels both in animal models and humans [517,518,519,520,521,522,523]. Moreover, acupuncture has also been shown to be effective in the management of cancer-related symptoms [524,525,526,527]. Specifically for somatostatin secretion induction, in rat and dog models, electroacupuncture has been demonstrated to increase serum somatostatin levels [528,529,530,531,532,533,534,535] and possibly SSTR expression in rabbits [536]. In humans, electroacupuncture and moxibustion could also influence serum somatostatin levels [537,538] and, in turn, it has been proposed that the endogenous levels of somatostatin may influence the effects of electroacupuncture [539,540].

Finally, as analyzed above, it is possible to use somatostatin agonists or antagonists, to improve imaging methods in certain cases. Another possibility nowadays is imaging-controlled biopsies such as Magnetic Resonance Imaging (MRI)-transrectal ultrasound fusion guided prostate biopsy [541,542]; maybe it is also possible to consider the use of radiolabelled agonists or antagonists for PET/Computed Tomography (CT)-guided biopsies, which have already entered clinical practice [543,544,545]. For a number of pathologies, like thymoma, MRI is considered to be the best imaging method [546,547], but perhaps the sensitivity of PET/CT could be augmented by using radiolabelled somatostatin analogs. Additionally, fluorescent somatostatin analogs were used in order to delineate tumor boundaries for SSTR_2_-expressing tumors during surgery [548]. This opens new research avenues for enhancing imaging techniques with clinical applications in oncology, by improving pre- and intraoperative as well as follow-up diagnostic performance [549,550,551]; these applications might also allow for improvements in robotic surgery [552,553]. Future enhancements may also include the combination of SSTR imaging with 3D printing in order to aid in tumor identification and separation [554,555,556,557].

There are several challenges to translating basic research on SSTRs into clinical practice. Firstly, the complexity of SSTR expression in various tumor types and between individuals can lead to inconsistent therapeutic responses [558,559]. Additionally, overcoming drug resistance caused by receptor desensitization or tumor adaptation needs further development of therapeutic agents or antagonists [560]. Lastly, translating results from preclinical animal studies to human patients may be hindered by the species-specific structure and function of SSTRs, leading to the necessity of significant phase II and III studies in order to establish therapeutic safety and efficiency [479].

## 6. Conclusions

The gradual introduction of SSTR antagonists into clinical trial status may reveal new intervention options. Despite a fair amount of research on the pathophysiological associations of somatostatin and its related drugs, open questions yet remain, which are interesting questions for further research.

While the interaction of somatostatin with its receptors is well documented, it remains to be seen if it, or its analogs, are able to interact with other extracellular or intracellular receptors and if such an interaction is associated with different effects. In that regard, the synthesis and use of chimeric somatostatin molecules seem an interesting research avenue. The existence of receptors sensitive to somatostatin and its analogs, other than the known SSTRs, can be explored in the context of therapeutic resistance exhibited by some cancers when treated with somatostatin analogs.

On the other hand, somatostatin antagonists, which are coming to the foreground of relevant medical research, should be examined for their potential to avert or mitigate the side effects of somatostatin agonists, without compromising their therapeutical potential.

Apart from all these considerations and intriguing research perspectives, the continued research on the properties and structure of SSTRs will, hopefully, facilitate the discovery of new, even more selective analogs, with higher therapeutic potential.

## Figures and Tables

**Table 1:** Basic properties and distribution of SSTRs.

Receptor	First Cloning	MR *	Length	Chromosomal Location	Major Tissue Sites	References
SSTR_1_	1992	45,000	391	14q13	Brain/CNS, gastrointestinal tract, pancreas	[27,28,29,30,31]
SSTR_2_	1992	41,305	369	17q24	Brain/CNS, gastrointestinal tract, pancreas, lymph tissue, adrenal glands	[27,32,33,34]
SSTR_3_	1992	46,000	418	22q13.1	Brain/CNS, pancreas, gastrointestinal tract, lymph tissue, adrenal glands	[35,36]
SSTR_4_	1993	45,000	388	20p11.2	Brain/CNS, retina, placenta	[32,37,38,39,40]
SSTR_5_	1994	39,000	364	16p13.3	Brain/CNS, pancreas, gastrointestinal tract, lymph tissue, adrenal glands, aortic smooth muscle cells, Sertoli cells	[5,41,42,43,44]

* MR refers to molecular weight/relative molar mass.

**Table 2:** Physiological functions of SSTR activation.

SSTR	Functions	References
SSTR_1_	Inhibition of GH, prolactin and calcitonin secretion, (possible) anti-inflammatory and anti-nociceptive, regulation of hippocampal function	[5,20,45]
SSTR_2_	Inhibition of gastrin, histamine, growth hormone, adrenocorticotropin, glucagon, insulin, TSH, interferon-γ secretion, modulation of eating and drinking behavior, inhibition of stress responses, antidepressant effects, retinal neuroprotection	[20,46,47,48,49,50,51,52]
SSTR_3_	Cell proliferation reduction and apoptosis induction, insulin release inhibition, growth hormone release inhibition	[20,53,54,55,56]
SSTR_4_	Learning and memory, locomotor activity increase, possible modulation of behavioral responses	[5,20,57,58]
SSTR_5_	Inhibition of growth hormone, adrenocorticotropin, insulin, glucagon-like peptide-1 and amylase secretion, (possible) anti-stress function, (possible) gastric emptying mediation	[5,20,59,60]

## Data Availability

Not applicable.
